# Effect of cryoprotectant concentration on bovine oocyte permeability and comparison of two membrane permeability modelling approaches

**DOI:** 10.1038/s41598-021-94884-0

**Published:** 2021-07-28

**Authors:** Tania García-Martínez, Teresa Mogas, Steven F. Mullen, Iris Martínez-Rodero, Ramila E. Gulieva, Adam Z. Higgins

**Affiliations:** 1 Department of Animal Medicine and Surgery, Autonomous University of Barcelona, 08193 Cerdanyola del Vallès, Spain; 2 COOK Medical, Bloomington, IN 47404 USA; 3 School of Chemical, Biological and Environmental Engineering, Oregon State University, Corvallis, OR 97331-2702 USA

**Keywords:** Biophysics, Cell biology, Developmental biology, Molecular biology

## Abstract

The plasma membrane permeability to water and cryoprotectant (CPA) significantly impacts vitrification efficiency of bovine oocytes. Our study was designed to determine the concentration-dependent permeability characteristics for immature (GV) and mature (MII) bovine oocytes in the presence of ethylene glycol (EG) and dimethyl sulphoxide (Me_2_SO), and to compare two different modeling approaches: the two parameter (2P) model and a nondilute transport model. Membrane permeability parameters were determined by consecutively exposing oocytes to increasing concentrations of Me_2_SO or EG. Higher water permeability was observed for MII oocytes than GV oocytes in the presence of both Me_2_SO and EG, and in all cases the water permeability was observed to decrease as CPA concentration increased. At high CPA concentrations, the CPA permeability was similar for Me_2_SO and EG, for both MII and GV oocytes, but at low concentrations the EG permeability of GV oocytes was substantially higher. Predictions of cell volume changes during CPA addition and removal indicate that accounting for the concentration dependence of permeability only has a modest effect, but there were substantial differences between the 2P model and the nondilute model during CPA removal, which may have implications for design of improved methods for bovine oocyte vitrification.

## Introduction

Over the last few decades, simultaneously with the development of assisted reproductive technologies, gamete and embryo cryopreservation procedures have advanced rapidly. These technologies have made a significant impact on the progress of genetic improvement in livestock, the worldwide distribution of germplasm and conservation of endangered species^1^. However, despite many offspring of various species being produced after the application of these technologies, there still remain shortcomings with methods used to cryopreserve oocytes^2,3^. The reason for oocytes susceptibility to low temperatures is due to their sensitivity at different cellular levels, such as the zona pellucida, plasma membrane, meiotic spindles and cytoskeleton (see review^4^). These subcellular structures change during maturation, which means that the developmental stage of the oocyte affects its cryobiological properties^5^. In addition, oocytes at different developmental stages have been shown to have different osmotic responses in the presence and absence of CPA^6,7^.

Vitrification has been proven to be more efficient and reliable than slow freezing for bovine oocyte cryopreservation because it resolves two of the main reasons for oocyte damage during slow freezing: chilling injury (by using high cooling and warming rates (typically >  > 100 °C/min)), and lethal ice crystal formation (by using high CPA concentrations (typically > 5 mol/L))^8–11^. CPAs have been demonstrated to dramatically suppress the freezing injuries suffered by cells. They promote the formation of a non-crystalline glassy state by increasing the viscosity of extra- and intracellular solutions and by interacting directly with water, which reduces ice nucleation and growth. CPAs are also beneficial by stabilizing the plasma membrane and reducing the harmful concentrated electrolytes through permeating into the cells^12^. However, there is a cost associated with their use as they dramatically increase the risk of damage due to osmotic stresses or death due to chemical toxicity^13,14^.

The process of adding and removing CPAs subjects the cells to an imbalanced osmotic pressure between the intra- and extracellular solutions. CPA addition results in cell shrinkage when water exits in response to the increased extracellular osmolality, and then re-swelling as the CPA and water permeate the cell returning the cell to isotonic volume. During CPA removal, the cell first swells to greater than isotonic volume as water moves into the cell and then returns to isotonic volume as CPA and water exit the cell^15^. These responses, if large enough, may drive the cell beyond critical volumes known as osmotic tolerance limits, outside of which irreversible cell damage occurs^16,17^. Typically, the use of stepwise addition and removal procedures can reduce concentration gradients enough to alleviate osmotic damage^18^.

It has been demonstrated that CPA toxicity is dependent on many factors including the CPA type, time of exposure to CPA, CPA concentration, and temperature^19–22^. The development of optimal cryopreservation protocols requires accounting for all interdependent factors. This makes rigorous experimental optimization impractical, as it would require a very large number of experiments. Therefore, it is desirable to combine empirical and theoretical knowledge through the use of mathematical modeling to simplify experimental optimization^23^. Frequently, the mathematical approaches rely on predictions of mass transfer across the cell membrane, which require an understanding of the cell membrane permeability to water (Lp) and CPA (Ps). Permeability parameters allow calculation of cellular osmotic responses during the addition and removal of CPA, and can provide evidence for whether water and solute movement occurs through channels or by simple diffusion through the lipid bilayer^24^. Recently, it has been shown that the permeability of the erythrocyte cell membrane to water and CPA is dependent on concentration^25^, contrary to what previous studies have assumed^26^.

Historically, the two-parameter formalism (2P model) has been used to model membrane transport^27,28^. This model makes limiting dilute-solution assumptions. The assumption of a dilute and ideal solution is often acceptable under physiological conditions, but its accuracy is questionable in most cryobiological cases where CPAs are often used at high concentrations. In 2009, Elmoazzen et al.^29^ developed a new nondilute solution model, which is comparatively more complex than the 2P model, but potentially more accurate.

The present study was designed to assess the potential inaccuracies of typical modeling approaches used for oocytes by examining concentration-dependent permeability characteristics for GV and MII bovine oocytes in the presence of EG or Me_2_SO, and comparing the two different mathematical approaches to mass transport modeling. To compare these different modeling approaches, we used the permeability parameters determined from the experiments to model cell volume excursions for the CPA addition and removal process used in the Kuwayama protocol^30^, which was designed for vitrification of bovine and human oocytes.

## Results

### Permeability parameters estimation

**Figure 1:**
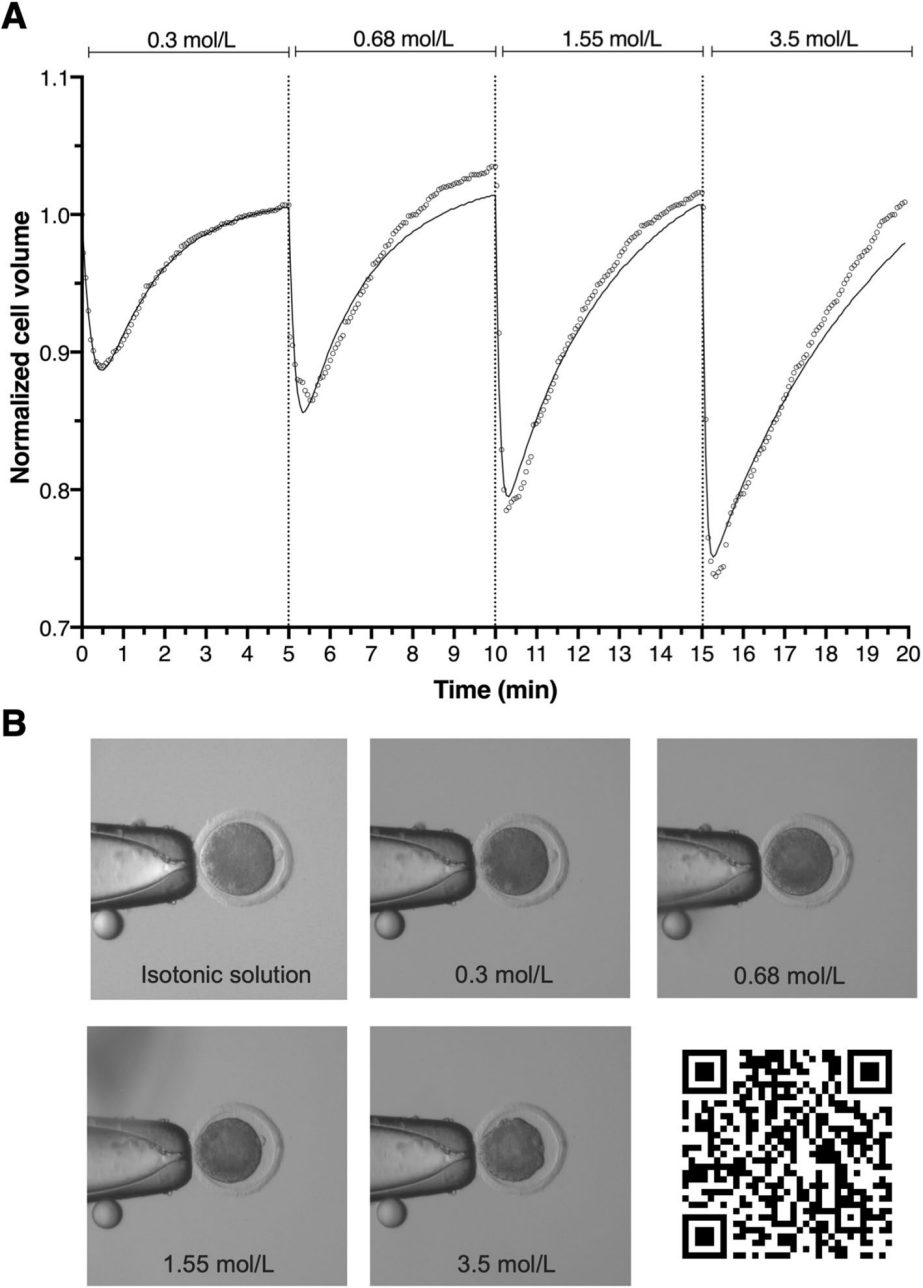
A representative sequence of the osmotic responses of MII bovine oocytes after exposure to increasing EG concentrations (0.3, 0.68, 1.55 and 3.5 mol/L). (**A**) The normalized cell volume data (open circle) and their corresponding theoretical fitting curves (solid line) obtained with the 2P model. Exposure to 0.3 mol/L EG starts at t = 0, and the change every 5 min to subsequent increasing EG concentrations is demarcated in the X-axis with a vertical dotted line. (**B**) An example of the oocyte’s morphology at relevant timeframes (at the minimal volume) is shown below each graphic (magnification 20x). Note shrinkage in response to increasing EG concentration. QR code links to a representative video time-lapse for an MII oocyte exposed to increasing EG concentrations.

To determine concentration-dependent permeability characteristics for GV and MII bovine oocytes, cells were sequentially exposed to a series of increasing CPA concentrations, as illustrated in Fig. 1. When cells were transferred from an isotonic solution into a hypertonic CPA solution they immediately dehydrated and shrank in response to the higher solution osmolality, and then slowly re-gained iso-osmotic volume as the solute and water permeated the cell to maintain osmotic equilibrium with the extracellular medium. The rate of this shrink-swell response is a measure of the permeability of the cell membrane to the solute and water^3^. As shown in Fig. 1, model predictions are in good agreement with the experimental cell volume measurements.

**Figure 2:**
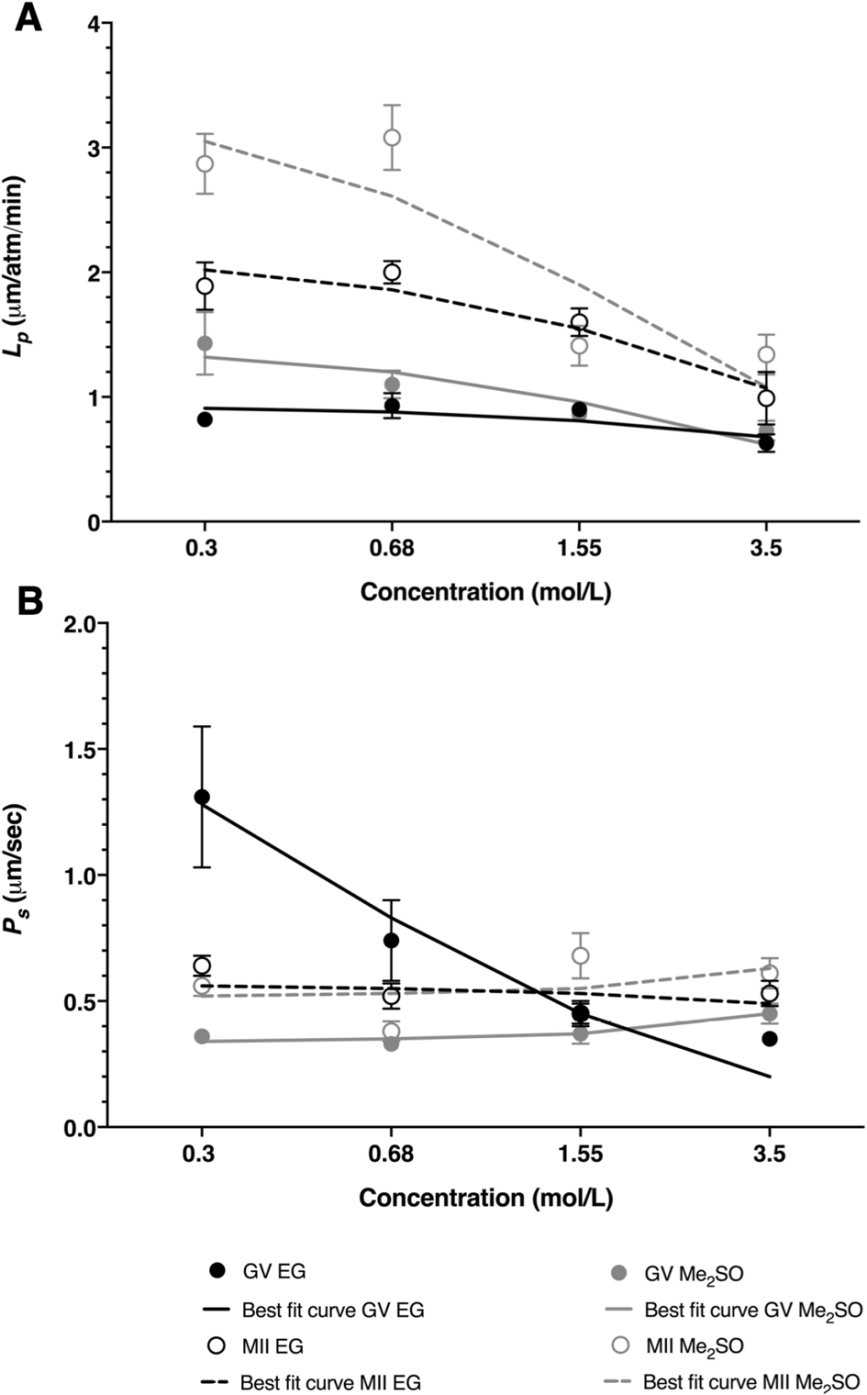
Water permeability and solute permeability GV (closed circles) and MII (open circles) oocytes in the presence of increasing concentration of CPA (EG (black) or Me_2_SO (gray)) for the 2P model (**A**, **B**, respectively). Best-fit curve for the concentration dependent model represented by a solid black line (GV EG), dotted black line (MII EG), solid gray line (GV Me_2_SO) and dotted gray line (MII Me_2_SO). Unless indicated otherwise, data are given as the mean ± s.e.m.

We first examined the potential differences in permeability values between GV and MII oocytes for exposure to EG and Me_2_SO. The resulting best-fit water and CPA permeability values from the 2P model are shown in Fig. 2 and Table 1. Overall, the water permeability was about two-fold higher for MII oocytes than GV oocytes. This was true for the water permeability in the presence of both Me_2_SO and EG, and the effect was statistically significant (p < 0.05). The CPA permeability was similar for Me_2_SO and EG, for both MII and GV oocytes, with the exception of the EG permeability for GV oocytes, which was substantially higher at low EG concentrations.

**Table 1:** Bovine oocyte plasma membrane water permeability (*L*_p_), solute permeability (*P*_s_) of immature (GV) and mature (MII) oocytes in the presence of increasing CPA (EG or Me_2_SO) concentrations.

	Stage	Solute	Concentration (mol/L)				Equation
			0.3	0.68	1.55	3.5	
*L*_p_ (µm/atm × min)	GV	Me_2_SO	1.43 ± 0.25^a,1^	1.10 ± 0.11^ab,1^	0.86 ± 0.06^ab,1^	0.73 ± 0.08^b,1^	*L*_p_ = 1.44/(0.29 × *M*_s_ + 1)
	MII	Me_2_SO	2.87 ± 0.24^a,2^	3.08 ± 0.26^a,2^	1.41 ± 0.16^b,2^	1.33 ± 0.16^b,2^	*L*_p_ = 3.51/(0.49 × *M*_s_ + 1)
	GV	EG	0.82 ± 0.05^ab,1^	0.93 ± 0.10^a,1^	0.90 ± 0.06^a,1^	0.63 ± 0.07^b,1^	*L*_p_ = 0.93/(0.08 × *M*_s_ + 1)
	MII	EG	1.89 ± 0.19^a,2^	2.00 ± 0.09^a,2^	1.60 ± 0.11^a,2^	0.99 ± 0.21^b,1^	*L*_p_ = 2.17/(0.24 × *M*_s_ + 1)
*P*_s_ (µm/sec)	GV	Me_2_SO	0.36 ± 0.03^a,1^	0.33 ± 0.03^a,1^	0.37 ± 0.04^a,1^	0.45 ± 0.04^a,1^	*P*_s_ = 0.34/(−0.05 × *M*_s_ + 1)
	MII	Me_2_SO	0.56 ± 0.04^ab,2^	0.38 ± 0.04^a,1^	0.68 ± 0.09^b,2^	0.61 ± 0.06^ab,1^	*P*_s_ = 0.51/(−0.04 × *M*_s_ + 1)
	GV	EG	1.31 ± 0.28^a,1^	0.74 ± 0.16^ab,1^	0.45 ± 0.05^b,1^	0.35 ± 0.03^b,1^	*P*_s_ = 2.18/(2.28 × *M*_s_ + 1)
	MII	EG	0.64 ± 0.04^a,1^	0.52 ± 0.05^a,1^	0.45 ± 0.04^a,1^	0.53 ± 0.05^a,2^	*P*_s_ = 0.57/(0.04 × *M*_s_ + 1)

Permeability parameters obtained from 0.3, 0.68, 1.55 and 3.5 mol/L EG or Me_2_SO data fit with the 2P model. The equation represents the best-fit model curve for each stage and CPA using the concentration dependent model described above. Unless indicated otherwise, data are given as the mean ± s.e.m. Different superscript letters indicate significant differences between different molarities within the same CPA and nuclear stage (*P* < 0.05). Different superscript numbers indicate significant differences in water or solute permeability between GV and MII stage within the same CPA (*P* < 0.05).

*GV* germinal vesicle, *MII* metaphase II, *Me*_*2*_*SO* dimethyl sulfoxide, *EG* ethylene glycol.

We next examined the potential effects of CPA concentration on the permeability values. Results indicate that CPA concentration had a statistically significant effect on the water permeability in all cases (p < 0.05), and, in general, the water permeability decreased by about a factor of two as the CPA concentration increased from 0.3 mol/L to 3.5 mol/L (Table 1).

Only GV oocytes exposed to EG exhibited a continuous decrease in CPA permeability with increasing CPA concentration (Table 1). In this case, the effect of CPA concentration was statistically significant (p = 0.002), and the CPA permeability decreased by more than threefold from 1.31 µm/s at 0.3 mol/L EG to 0.35 µm/s at 3.5 mol/L EG. The effect of CPA concentration was also significant for MII oocytes exposed to Me_2_SO (p = 0.014), but in this case, the CPA permeability did not exhibit a clear trend: the lowest permeability was 0.38 µm/s at 0.68 mol/L and the highest was 0.68 µm/s at 1.55 mol/L.

**Figure 3:**
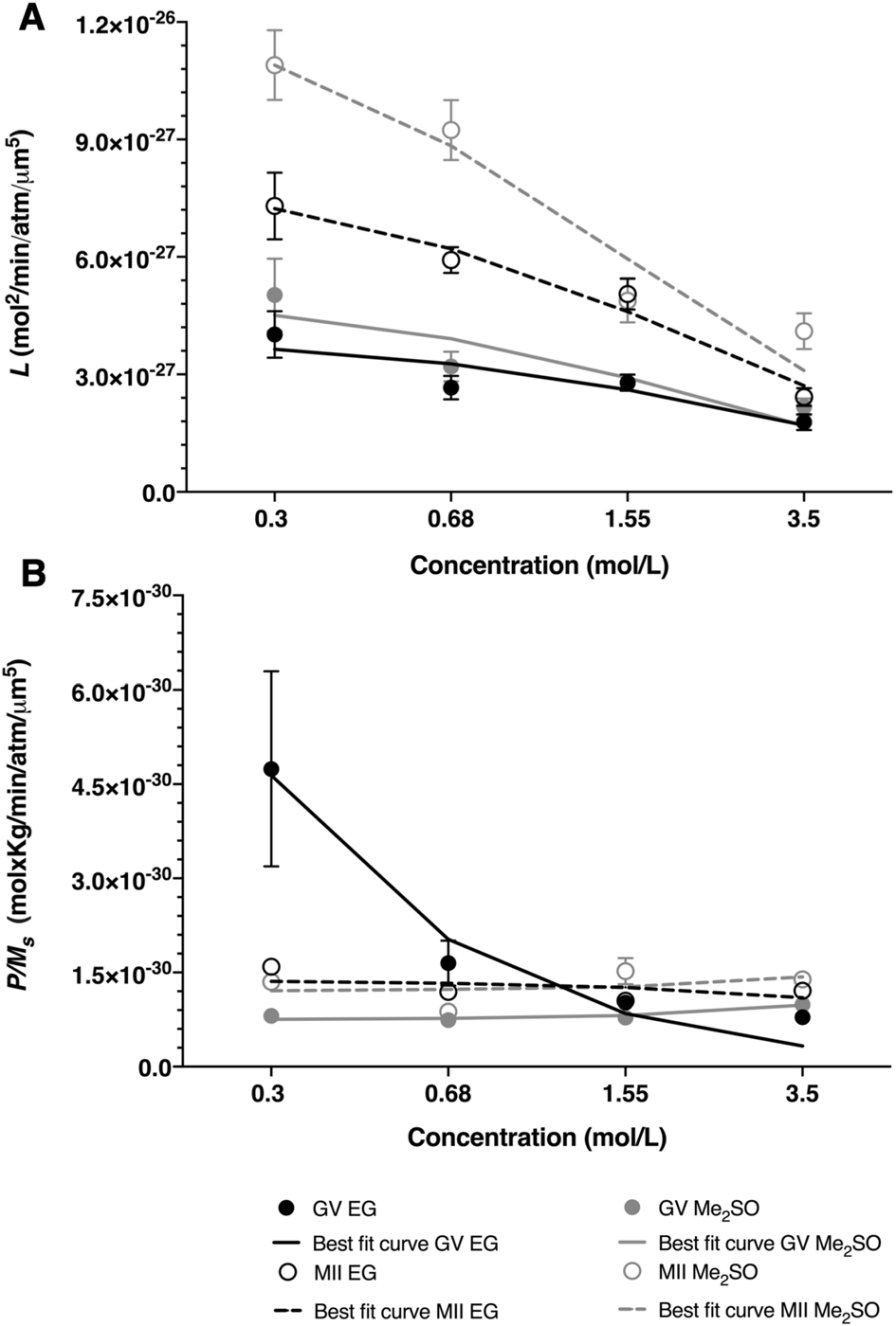
Water permeability and solute permeability GV (closed circles) and MII (open circles) oocytes in the presence of increasing concentrations of CPA (EG (black) or Me_2_SO (gray)) for the nondilute solution model (**A**, **B**, respectively). Best-fit curve for the concentration dependent model is represented in solid black line (GV EG), dotted black line (MII EG), solid gray line (GV Me_2_SO) and dotted gray line (MII Me_2_SO). Unless indicated otherwise, data are given as the mean ± s.e.m.

We also fit the data to the non-dilute model and analyzed the resulting best-fit permeability parameters. As shown in Fig. 3 and Table 2, the trends for the non-dilute model were nearly identical to those observed for the 2P model. Similar to the water permeability from the 2P model, the best-fit water permeability values from the non-dilute model decreased by a more than a factor of two as the CPA concentration increased from 0.3 mol/L to 3.5 mol/L, and the effect of CPA concentration on the water permeability was statistically significant in all cases (p < 0.004). For the non-dilute model, only the CPA permeability parameter for GV oocytes exposed to EG showed a continuous decrease with increasing concentration, which is consistent with the results for the 2P model. However, there was an even more substantial decrease in the CPA permeability parameter for the non-dilute model, which yielded a CPA permeability parameter at 3.5 mol/L that was about six times lower than the permeability parameter at 0.3 mol/L. Only the oocytes at the GV stage exposed to Me_2_SO showed apparent constant permeability to that cryoprotectant across all concentrations. The oocytes in the other categories did exhibit apparent changes to CPA permeability between different concentrations, but the trend was not consistent.

**Table 2:** Bovine oocyte plasma membrane water permeability (*L*), solute permeability (*P*) of immature (GV) and mature (MII) oocytes in the presence of increasing CPA (EG or Me_2_SO) concentrations.

	Stage	Solute	Concentration (mol/L)				Equation
			0.3	0.68	1.55	3.5	
*L* × 10^–27^ (mol^2^/min/atm/µm^5^)	GV	Me_2_SO	5.03 ± 0.93^a,1^	3.20 ± 0.38^ab,1^	2.77 ± 0.20^b,1^	2.17 ± 0.21^b,1^	*L* = 5.13/(0.44 × *M*_s_ + 1)
	MII	Me_2_SO	10.88 ± 0.89^a,2^	9.24 ± 0.76^a,2^	4.88 ± 0.56^b,2^	4.10 ± 0.46^b,2^	*L* = 13.34/(0.71 × *M*_s_ + 1)
	GV	EG	4.02 ± 0.59^a,1^	2.66 ± 0.30^ab,1^	2.79 ± 0.21^ab,1^	1.78 ± 0.20^b,1^	*L* = 3.98/(0.31 × *M*_s_ + 1)
	MII	EG	7.30 ± 0.85^a,2^	5.92 ± 0.33^a,2^	5.05 ± 0.40^a,2^	2.42 ± 0.23^b,1^	*L* = 8.28/(0.47 × *M*_s_ + 1)
*P*/*M*_s_ × 10^–30^ (mol × Kg/min/atm/µm^5^)	GV	Me_2_SO	0.81 ± 0.07^a,1^	0.74 ± 0.07^a,1^	0.78 ± 0.09^a,1^	0.99 ± 0.09^a,1^	*P*/*M*_s_ = 0.74/(−0.05 × *M*_s_ + 1)
	MII	Me_2_SO	1.35 ± 0.11^a,2^	0.88 ± 0.09^b,1^	1.52 ± 0.21^a,2^	1.39 ± 0.12^a,2^	*P*/*M*_s_ = 1.20/(−0.04 × *M*_s_ + 1)
	GV	EG	4.74 ± 1.55^a,1^	1.65 ± 0.36^ab,1^	1.02 ± 0.12^b,1^	0.78 ± 0.07^b,1^	*P*/*M*_s_ = 43.1/(30 × *M*_s_ + 1)*
	MII	EG	1.59 ± 0.12^a,1^	1.19 ± 0.12^b,1^	1.05 ± 0.10^b,1^	1.21 ± 0.11^ab,2^	*P*/*M*_s_ = 1.38/(0.06 × *M*_s_ + 1)

Permeability parameters obtained from 0.3, 0.68, 1.55 and 3.5 mol/L EG or Me_2_SO data fit with the nondilute model. The equation represents the best-fit model curve for each stage and CPA. Unless indicated otherwise, data are given as the mean ± s.e.m. Different superscript letters indicate significant differences between different molarities within the same CPA and nuclear stage (*P* < 0.05). Different superscript numbers indicate significant differences in water or solute permeability between GV and MII stage within the same CPA (*P* < 0.05). * In this case the fitting algorithm was unable to converge because the value of *b* was so large that the 1 in the denominator became negligible. This results in a constant ratio *a*/*b*, which can be satisfied using various combinations of *a* and *b*. The values of a and b in Table 2 were chosen arbitrarily among the possible values that match the best-fit value of *a*/*b*.

*GV* germinal vesicle, *MII* metaphase II, *Me*_*2*_*SO* dimethyl sulfoxide, *EG* ethylene glycol.

To account for the effects of CPA concentration on water and CPA permeability, we fit the permeability data to a concentration-dependent model^25^. This model is consistent with a transport mechanism that is limited by binding of CPA to a transporter protein such as an aquaporin. The resulting model fits are shown as lines in Figs. 2 and 3, and the best-fit equations are provided in Tables 1 and 2. The concentration dependent model fits are in reasonable agreement with the permeability data.

### Model comparison

**Figure 4:**
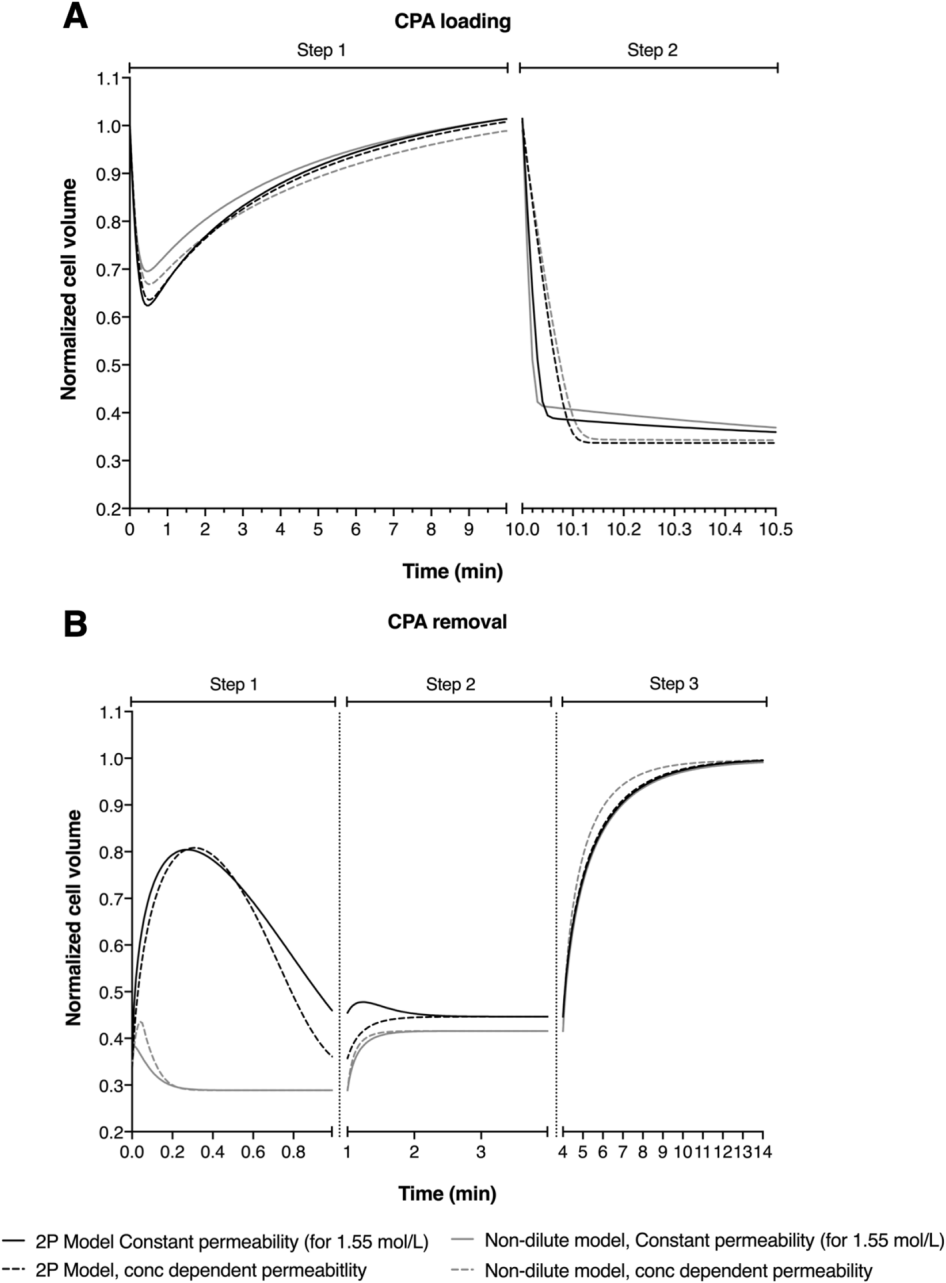
Comparative simulation of cell volume excursion of GV-stage bovine oocytes during CPA addition (**A**) and removal (**B**) following the Kuwayama protocol for the 2P model (black line) and nondilute model (gray line) using constant (solid line) and non-constant permeability parameters (dotted line).

**Figure 5:**
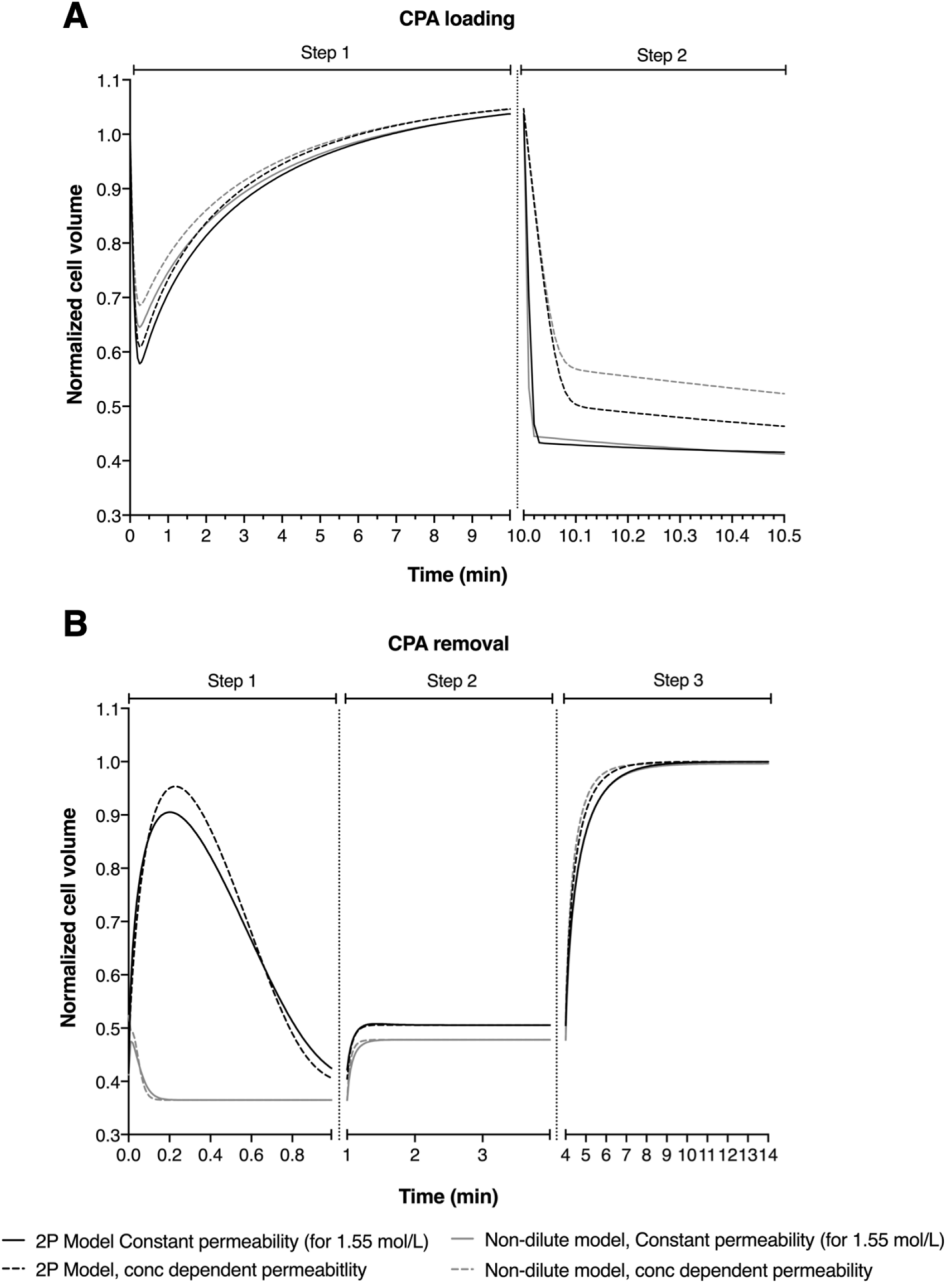
Comparative simulation of cell volume excursion of MII-stage bovine oocytes during CPA addition (**A**) and removal (**B**) following the Kuwayama protocol for the 2P model (black line) and nondilute model (gray line) using constant (solid line) and non-constant permeability parameters (dotted line).

To examine the potential practical implications of different modeling approaches, we simulated the cell volume response of both GV and MII bovine oocytes during CPA addition and removal following the Kuwayama vitrification protocol^30^, which was designed for and tested on bovine and human oocytes (see materials and methods for more details). Figures 4 and 5 show predictions for GV and MII oocytes for four different modeling approaches.

The most common approach for predicting cell volume changes during CPA addition and removal is to use the 2P model with constant water and CPA permeability values. This baseline case is shown by the solid black lines in Figs. 4 and 5. For comparison, the dotted black lines show predictions for the 2P model using concentration dependent permeability values. The two modeling approaches yield nearly identical predictions, and the slight differences are small and not likely to have practical significance. The non-dilute model predictions are also similar to the 2P model baseline case, with the exception of the first step of CPA removal. The non-dilute model exhibits less swelling and faster equilibration during the first step of CPA removal than the 2P model.

## Discussion

The development of a reliable method for the cryopreservation of mammalian oocytes is crucial for assisted reproduction in both human and domestic species^31–33^. However, in bovine species, the success rates are still limited due to the oocytes’ unique structure and sensitivity to cooling^4,34^. One of the most important cryobiological properties that affects the survival of a cell after vitrification is the permeability of the plasma membrane to water and CPA^35^. These permeability values determine the extent of cell volume changes and the time required for CPA equilibration. Therefore, knowledge of these permeability parameters is useful for predicting the likely optimal conditions during CPA addition and removal. Typically cell membrane permeability parameters are determined for exposure to a single CPA concentration^3,6,36^. However, oocytes are exposed to various CPA concentrations during the vitrification process, and previous studies suggest that the water and CPA permeability may be concentration dependent^25^. Therefore, in this study, we examined the potential effects of CPA concentration on the membrane permeability parameters and the implications for bovine oocyte vitrification.

Table 3 shows the water and CPA permeability values for bovine oocytes determined in previous studies ^3,6,36^. In these studies, the permeability parameters were estimated by measuring cell volume changes after exposure to a single CPA concentration ranging between 1.2 mol/L and 1.8 mol/L. In the current study, we measured the permeability for various CPA concentrations by sequentially exposing oocytes to increasing concentrations, but the most appropriate concentration for direct comparison to previous studies is 1.55 mol/L (see Table 1). Overall, our results are consistent with the trends observed in previous studies: the water permeability of MII oocytes was nearly twofold higher than that of GV oocytes, and the CPA permeability was similar for EG and Me_2_SO and for GV and MII oocytes. However, the water permeability values determined in this study were higher than previous studies. The reason for this discrepancy is unclear but may be related to differences in the methods used to determine the permeability values^37,38^.

**Table 3:** Previously determined bovine oocyte plasma membrane water permeability (Lp) and cryoprotectant permeability (Ps) values in the presence of CPA at room temperature.

CPA	Molarity (mol/L)	Stage	*L*_p_ (μm/min/atm)	*P*_s_ (μm/sec)	References
Me_2_SO	1.5	GV	0.69*	0.37*	Agca et al., 1993^6^
EG	1.5	GV	0.50*	0.22*	Agca et al., 1993^6^
Me_2_SO	1.5	MII	1.16*	0.49*	Agca et al., 1993^6^
EG	1.5	MII	0.76*	0.42*	Agca et al., 1993^6^
EG	1.8	GV	0.11	0.55	Wang et al.^3^
EG	1.8	MII	0.20	0.53	Wang et al.^3^
Me_2_SO	1.2	MII	1.24	0.25	Jin et al. ^36^
EG	1.3	MII	0.84	0.58	Jin et al. ^36^

GV, germinal vesicle; MII, metaphase II; EG, ethylene glycol; Me_2_SO, dimethyl sulfoxide.

*Published *L*_p_ and *P*_s_ values were determined using the Kedem-Katchalsky (KK) model^54^. These KK parameters were converted to the corresponding *L*_p_ and *P*_s_ values for the 2P model as described previously^55^.

Our results demonstrate that the water permeability of bovine oocytes decreases as the CPA concentration increases. This trend has been observed previously for various other cell types^25,39,40^, and has been explained in terms of steric hindrance caused by CPA binding to water-transporting channels^41^. Bovine oocytes are known to express aquaporins^36,42^ and the observation that water permeability decreases as CPA concentration increases is consistent with such a mechanism. For some cell types, including mouse oocytes, the water permeability has been observed to increase as CPA concentration increases^37,38^. The reason for this trend is unclear, but it does not appear to be relevant to bovine oocytes.

Our results also show that the EG permeability of bovine GV oocytes decreases as EG concentration increases. Bovine oocytes express both aquaporin 3 and aquaporin 7^42^. These aquaglyceroporins have been shown to transport glycerol, ethylene glycol and possibly other CPAs^36^ and are postulated to transport these molecules via successive binding to various sites on the aquaporin protein^43,44^. These binding sites can become saturated at high CPA concentrations, leading to slower CPA transport^43,44^. In contrast to glycerol and ethylene glycol, there is evidence that Me_2_SO transport through aquaporin 3 is negligible in mammalian oocytes^35^, which may explain why we did not observe a concentration dependence for the Me_2_SO permeability. We also did not observe a concentration dependence of the EG permeability for MII oocytes, suggesting that changes in aquaporin expression or membrane composition during oocyte development may have impacted the EG transport mechanism^45^.

One potential explanation for the observed effects of CPA concentration on the permeability parameters is the use of the 2P model for making predictions under non-dilute conditions. It has been suggested that the non-dilute transport model (Eqs. 5 and 6) can provide more accurate predictions, particularly at high CPA concentrations^29^. Therefore, we also analyzed the permeability parameters for GV and MII bovine oocytes with the non-dilute transport model^29^. Our results show nearly identical tendencies with both mathematical models: the water permeability decreases as EG or Me_2_SO concentration increases in both GV and MII oocytes, and the EG permeability decreases as EG concentration increases in GV oocytes (see Tables 1 and 2). Elmoazzen et al.^29^ argued that when the permeability coefficient P is divided by concentration (as we have done in Table 2), the resulting ratio should not be dependent on CPA concentration. Nevertheless, our results indicate that GV oocytes exposed to EG exhibit a substantial decrease in the value of this ratio as the EG concentration increases. This indicates that the observed decreases in water and CPA permeability with increasing CPA concentration cannot be attributed only to use of the 2P model under non-dilute conditions, suggesting a physical transport process that is CPA concentration dependent.

Cell membrane transport predictions such as those shown in Fig. 5 can be useful for evaluating the potential for damage during CPA addition and removal and for design of less damaging methods. Many cell types have limited tolerance for changes in cell volume, with increasing volume changes causing additional loss of cell viability^14,17^. Thus, knowing the osmotic tolerance limits is essential for optimizing cryopreservation methods. Previously, Mullen et al.^46^ demonstrated that MII spindle damage in bovine oocyte occurs more frequently as the extracellular solution concentrations diverge from isosmotic. Moreover, they estimated that to prevent osmotic damage to the MII spindle with a probability of 90%, it is necessary to use CPA addition and removal procedure which maintains the cells within a volume range of 1.1 to 0.52 times the isotonic volume. Therefore, using the Kuwayama protocol^30^ for bovine oocytes is not expected to cause very much osmotic damage to the spindle during CPA loading based on predictions using any of the modeling approaches that we investigated (see Fig. 5). However, during CPA removal, the non-dilute model predicts that GV and MII oocytes will shrink to an equilibrium value much faster than the 2P model predicts, which involves maintaining the cells in a more prolonged state of osmotic stress. Based on the osmotic damage model developed by Mullen et al.^46^, maintenance of the oocytes at a volume of 38% relative to isotonic is expected to cause about 35% of oocytes to experience osmotic damage to the spindle.

Exposure to CPA can also cause cell damage due to toxicity. Although toxicity is expected to be less of a problem during CPA removal, it is still important to design removal procedures with toxicity in mind. Benson et al.^47^ demonstrated that inducing swelling is beneficial because it decreases the intracellular CPA concentration and its associated toxicity. Human oocytes swell to more than their isotonic volume during the first step of the CPA removal process after vitrification^48^. In contrast, bovine oocytes are predicted to exhibit much less swelling (Figs. 4 and 5), especially for the non-dilute model, which may increase CPA toxicity.

Overall, the predictions presented in Fig. 5 suggest that modifying the first step of the CPA removal process may reduce damage to bovine oocytes. In particular, we observed that the non-dilute model predicts that the amount of osmotic swelling after vitrification relative to the starting (shrunken) volume is minor compared to the dilute model. To our knowledge, we are the first to demonstrate that the non-dilute model and dilute model predict such different volume responses during CPA removal, highlighting the need for future studies to examine the relative accuracy of the two different modeling approaches. If the non-dilute model predictions turn out to be more accurate than those from the dilute model, this suggests that a very different protocol for CPA removal should be well tolerated (a lot less sucrose would be needed as an osmotic buffer in step 1). By reducing the amount of sucrose in the medium the oocytes would not be expected to shrink as much, keeping them within osmotic tolerance limits. This is expected to cause much less damage than the original protocol^48^.

## Conclusions

Historically, cell permeabilities to water and CPA were assumed to be independent of CPA concentration. This is likely due to the predominance of slow cooling methods employed for cellular cryopreservation for the last half century, where a single concentration of CPA was used. In recent years, vitrification has become the preferred method for mammalian oocyte cryopreservation, requiring reconsideration of this assumption. In this study, we have examined the effects of CPA concentration on water and CPA membrane permeability for bovine oocytes. We have shown that water permeability is inversely related to CPA concentration. Furthermore, CPA concentration also affects membrane CPA permeability, with differential effects depending upon the maturation stage of the oocyte and the specific CPA type. Although both the water and CPA permeability change with concentration, accounting for the concentration dependence of permeability only had a slight effect on cell volume predictions during CPA addition and removal, suggesting that the typical assumption that permeability is independent of concentration is reasonable. We have also investigated two modeling approaches, one using dilute solution assumptions, and another that is not restricted to those assumptions. The results suggest that only slight differences exist in the predictions during the CPA loading steps of the procedure, but a greater difference was noted between the two models’ predictions during the first stage of CPA removal. This may have important implications for developing improved procedures for the vitrification of mammalian oocytes.

## Material and method

### Reagents

Unless otherwise specified, all chemicals and reagents were purchased from Sigma Chemical Co (St. Louis, MO, USA).

### Oocyte collection and in vitro maturation

The in vitro maturation (IVM) procedure followed has been described previously^49^. Briefly, ovaries from slaughtered postpubertal heifers (12–18 months old) were transported from a local slaughterhouse to the laboratory in saline solution (0.9% NaCl) at 35–37 °C within 2 h. Immature cumulus-oocyte complexes (COCs) were aspirated from 3 to 8 mm follicles using an 18-gauge needle attached to a 5 mL syringe. COCs with more than three layers of cumulus cells and a homogeneous cytoplasm were selected and washed three times in modified Dulbecco’s PBS (PBS supplemented with 36 µg/mL pyruvate, 50 µg/mL gentamicin and 0.5 mg/mL bovine serum albumin). Groups of 40–50 COCs were placed in 500 µL of maturation medium covered with mineral oil in four-well plate and cultured for 24 h at 38.5 °C in a 5% CO_2_ humidified air atmosphere. The maturation medium (IVM medium) was tissue culture medium (TCM-199) supplemented with 10% (v/v) fetal bovine serum (FBS), 10 ng/mL epidermal growth factor and 50 µg/mL gentamicin.

### Measurement of oocyte volumetric changes following increasing CPA exposure

**Figure 6:**
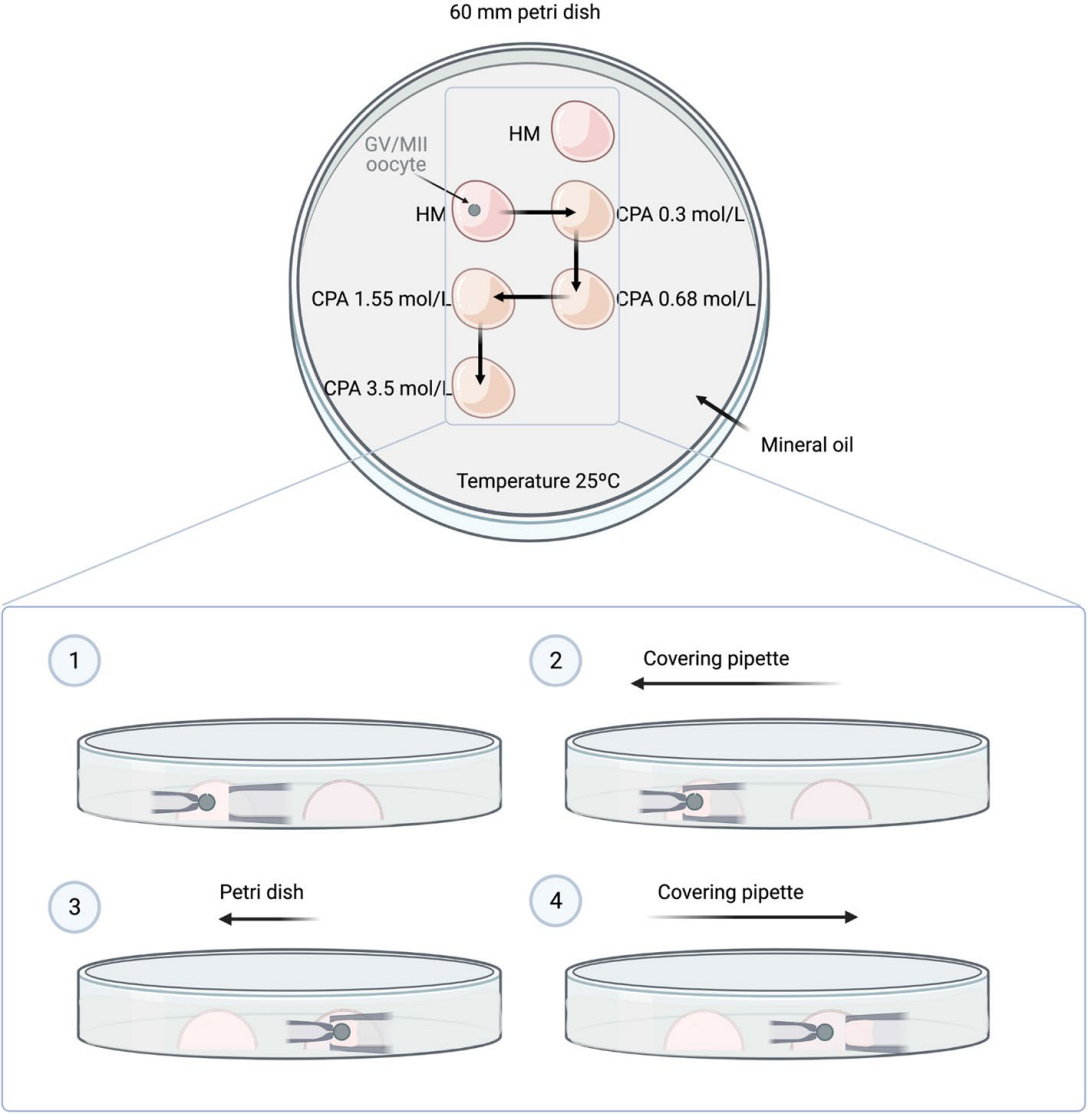
Schematic representation of the device and the procedure for direct transfer of GV or MII oocytes (solid circle) to an increasing CPA exposure (right hemisphere) from isotonic holding medium (left hemisphere) using a micromanipulator system.

GV bovine oocytes at time 0 h or MII bovine oocytes after 24 h of IVM were denuded of cumulus cells by gentle pipetting. Only GV showing a normal appearance and metaphase II oocytes with a normal appearance and a visible first polar body were used. An oocyte was placed in a 25 µl drop of holding medium (HM: TCM199-Hepes supplemented with 20% (v/v) FBS) covered with mineral oil, and was held with a holding pipette (outer diameter, 95–120 µm; MPH-MED-30, Origio, Denmark) connected to a micromanipulator on an inverted microscope (Zeiss Axio Vert A1, Germany). An initial photograph was taken of the oocyte in order to calculate the initial volume. The oocyte was then covered with another pipette with a larger inner diameter (600-µm diameter) (G-1 Narishigue, Tokyo, Japan) connected to a different micromanipulator. Then, by sliding the dish the oocyte was exposed consecutively to 25 µl drops containing increasing CPA concentrations at 25 °C, as illustrated in Fig. 6. Each oocyte was exposed consecutively to CPA concentrations of 0.30 mol/L, 0.68 mol/L, 1.55 mol/L, and 3.5 mol/L. These concentrations were chosen because they are expected to generate similar cell volume changes for each change in CPA concentration. In addition, the gradual increase in CPA concentration prevents excessive shrinkage which may result in osmotic damage. For the exposure times at each concentration it was considered the time needed for the oocyte to recover the isotonic cell volume and was set at 5 min for EG and 7 min for Me_2_SO. CPA solutions consisted of EG or Me_2_SO diluted in HM. The cell volume response of the oocyte during the experiments was recorded every 3.5 s with a time-lapse video recorder (Zeiss Zen imaging software/Axiocam ERc 5 s). The volume of the oocyte in each image was calculated from the area of the cross section using ImageJ software. Only those immature oocytes (EG: n = 6; Me_2_SO: n = 5) and mature oocytes (EG: n = 5; Me_2_SO: n = 7) that remained spherical on shrinkage were individually analyzed and used for calculation of permeability coefficients, with several oocytes in each group being discarded.

### Membrane transport models

#### Dilute solution model (two-parameter transport formalism)

The 2P model, which has its roots in work by Jacobs and Stewart^27,28^, provides a description of the osmotic responses of cells in solutions with both permeating and nonpermeating solutes. In this formalism, the water flux into the cell over time is expressed as:

1 $$\frac{d{V}_{w}}{dt}=-{L}_{p}ART({M}^{e}-{M}^{i})$$

where *V*_w_ is the cell water volume, *L*_p_ is the membrane hydraulic conductivity, *A* is the area of the plasma membrane, *R* is the universal gas constant, *T* is the absolute temperature, and *M*^e^ and *M*^i^ are the total external and internal osmolalities, respectively.

The rate of CPA transport is given by:

2 $$\frac{d{N}_{s}}{dt}={P}_{s}A({M}_{s}^{e}-{M}_{s}^{i})$$

where *N*_s_ is the intracellular moles of CPA, *P*_s_ is the CPA permeability, *M*^i^_s_ and *M*^e^_s_ are the intracellular and extracellular CPA molality, respectively. To obtain the intracellular CPA volume, it is necessary to multiply by the partial molar volume of the CPA, υ_s_, resulting in

3 $${V}_{s}={\upsilon }_{s}{N}_{s}$$

Then the total cell volume (*V*_c_) is just the sum of the water (*V*_w_), CPA (*V*_s_), and solids (*V*_b_) volumes:

4 $${V}_{c}={V}_{w}+{V}_{s}+{V}_{b}$$

#### Nondilute solution model

Elmoazzen et al.^29^ developed the nondilute solution model. The transport equations are based on the principle that the mass transfer is driven by the difference between the extra- and intracellular chemical potentials. This model was developed on the assumption that the extracellular solution contains water, permeating CPA and a nonpermeating solute (NaCl), while the intracellular environment contains water, a permeating CPA, and another nonpermeating solute (KCl). The changes in the moles of intracellular water and CPA as a function of time take the following forms:

5 $$\frac{d{N}_{w}^{i}}{dt}=-LART\left[\left({x}_{CPA}^{e}+{x}_{NaCl}^{e}\right)+{B}_{CPA}^{+}\left({x}_{CPA}^{e}\right)+{B}_{NaCl}^{+}\left({x}_{NaCl}^{e}\right)+{(B}_{CPA}^{+}+\,{B}_{NaCl}^{+}){x}_{CPA}^{e}{x}_{NaCl}^{e}-\left({x}_{CPA}^{i}+{x}_{KCl}^{i}\right)-{B}_{CPA}^{+}\left({x}_{CPA}^{i}\right)-{B}_{KCl}^{+}\left({x}_{KCl}^{i}\right)-{(B}_{CPA}^{+}+{B}_{KCl}^{+}){x}_{CPA}^{i}{x}_{KCl}^{i}\right]$$

6 $$\frac{d{N}_{CPA}^{i}}{dt}=PART\left[ln\left({x}_{CPA}^{e}\right)+{(\frac{1}{2}-B}_{CPA}^{+})\left(1-{x}_{CPA}^{e}-{x}_{NaCl}^{e}\right)\left(1-{x}_{CPA}^{e}\right)-(\frac{1}{2}-{B}_{NaCl}^{+})\left(1-{{x}_{CPA}^{e}-x}_{NaCl}^{e}\right){x}_{NaCl}^{e}-ln\left({x}_{CPA}^{i}\right)-{(\frac{1}{2}-B}_{CPA}^{+})\left(1-{x}_{CPA}^{i}-{x}_{KCl}^{i}\right)\left(1-{x}_{CPA}^{i}\right)+(\frac{1}{2}-{B}_{KCl}^{+})\left(1-{{x}_{CPA}^{i}-x}_{KCl}^{i}\right){x}_{KCl}^{i}\right]$$

### Estimation of cell membrane permeability parameters

A randomized block design was used for this experiment, with the oocyte being the blocking factor^50^. Each oocyte was used in the entire series of solutions for a single CPA. In other words, an oocyte was used to estimate the permeability of the CPA at each concentration, but for only one of the two CPAs. Volumetric data for each oocyte at each concentration was assessed as described and the first 3 min were fitted to the 2P and nondilute solution models to determine the water permeability (*L*_p_ and *L*, respectively) and CPA permeability (*P*_CPA_ and *P*, respectively). This was performed for both GV and MII oocytes. The differential equations (Eqs. 1 and 2) for the 2P model; Eqs. (5) and (6) for the nondilute solution model) were solved in Matlab software using the ode45 function, which implements an explicit Runge–Kutta formula^51,52^. To estimate the permeability values, model predictions were fit to the data by minimizing the sum of the error squared in Matlab using the fminsearch function, which implements the Nelder-Mead simplex algorithm^53^. For the first CPA concentration, the initial state was assumed to be the normal physiological state for oocytes in equilibrium with isotonic solution. For subsequent CPA concentrations, the initial state was assumed to be equal to the final state from model predictions for the previous CPA concentration. The concentrations used in these experiments are given in Table 4 in units of molarity, molality and mole fraction. The constants used for model predictions are given in Table 5.

**Table 4:** CPA concentration.

CPA	Molarity (M)	Molality (m)	Mole fraction (X^e^_CPA_)
EG	0.3	0.31	0.06
	0.68	0.71	0.013
	1.55	1.7	0.030
	3.5	4.36	0.074
Me_2_SO	0.3	0.3	0.06
	0.68	0.71	0.013
	1.55	1.74	0.031
	3.5	4.64	0.078

*CPA* cryoprotectant, *EG* ethylene glycol, *Me*_*2*_*SO* dimethyl sulfoxide.

**Table 5:** Constant and parameters used in 2P model and nondilute solution model.

Description	Values	Symbol
Universal gas constant 2P model Nondilute model	8.314 m^3^ Pa K^-1^ mol^−1^ 8.206 × 10^13^ μm^3^ atm mol^−1^ K^−1^	R
Absolute temperature	298 K	T
Partial molar volume of water	18.02 × 10^12^μm^3^mol^−1^	υ_w_
Partial molar volume of CPA EG^a^ Me_2_SO^a^	55.8 × 10^–6^ m^3^ mol^−1^ 71.3 × 10^–6^ m^3^ mol^−1^	υ_CPA_
Osmotically inactive volume GV MII	0.16^3,7^ 0.25^7,36^	V_b_
Second osmotic virial coefficient for CPA (in terms of mole fraction) EG^b^ Me_2_SO^b^ Sucrose^b^	3.41 2.35 8.68	B^+^_CPA_
Second osmotic virial coefficient for NaCl (in terms of mole fraction)^b^ (Dissociation constant)^b^	3.80 1.644	B^+^_NaCl_
Second osmotic virial coefficient for KCl (in terms of mole fraction)^b^ (Dissociation constant)^b^	0 1.818	B_KCl_
Extracellular salt (NaCl) mole fraction	0.003	x^+^^e^_NaCl_
Extracellular salt (KCl) mole fraction	0.0030	x ^i^_KCl_

^a^Partial molar volumes of cryoprotectants from Vian et al.^56^.

^b^ Second osmotic virial coefficient and dissociation constants from Zielinski et al.^57^. Abbreviations: *CPA* cryoprotectant, *EG* ethylene glycol, *Me*_*2*_*SO* dimethyl sulfoxide, *GV* germinal vesicle, *MII* metaphase II.

To characterize the effects of CPA concentration, the permeability data was fit to the following concentration-dependent permeability models^25^

7 $${L}_{p}=\frac{a}{b{M}_{s}+1}$$

8 $${P}_{s}=\frac{a}{b{M}_{s}+1}$$

9 $$L=\frac{a}{b{M}_{s}+1}$$

10 $$\frac{P}{{M}_{s}}=\frac{a}{b{M}_{s}+1}$$

where *a* and *b* are best-fit constants. In Eq. (10), we use the ratio of the non-dilute model permeability coefficient *P* to the CPA molality *M*_s_, for the reasons described in Elmoazzen et al.^29^. In particular, Elmoazzen et al.^29^ show that the nondilute model reduces to the 2P model under dilute conditions, where the parameter ratio *P*/*M*_CPA_ is proportional to the 2P model permeability *P*_CPA_.

### Prediction of cell volume changes during bovine oocyte vitrification

The following Kuwayama protocol^30^ for CPA addition and removal for vitrification of oocytes was used to predict the response of oocytes and compare different modeling approaches:

Two steps for loading CPA:

- *Step 1* 10 min in 1.6 mol/L EG in TCM199 medium at 22°C.
- *Step 2* 30 s in vitrification solution (6.8 mol/L EG + 1.0 mol/L sucrose in TCM199 medium).

Three steps for removal CPA:

- *Step 1* 1 min in 1.0 mol/L of sucrose in TCM199 medium at 37°C.
- *Step 2* 3 min in diluent solution (0.5 mol/L sucrose in TCM199 medium)
- *Step 3* 10 min in TCM199 medium (no CPA).

We compared volume excursion predictions during CPA addition and removal using four different modeling approaches:

1. Dilute (2P) model with constant water and CPA permeability.
2. Dilute (2P) model with water and CPA permeability changed with CPA concentration according to equations (7) and (8).
3. Nondilute model with constant water and CPA permeability.
4. Nondilute model with water and CPA permeability changed with CPA concentration according to equations (9) and (10).

Predictions for the CPA addition and removal process following these 4 conditions were carried out by numerically solving the model equations in Matlab as described above. To obtain predictions with constant permeability values, we used the permeability to water and EG obtained at 1.55 mol/L. This represents a baseline case that is consistent with the typical approach of estimating permeability for exposure to 1–2 mol/L CPA. To make predictions using concentration dependent permeability values, we used a different approach during CPA addition and removal. For CPA addition, we used the external CPA concentration in Eqs. (7–10) to estimate the permeability values. For CPA removal, we used the intracellular CPA concentration to estimate the permeability values.

In order to simulate the cell volume response of the vitrification and warming Kuwayama’s protocol, information on the isotonic cell volume of bovine oocytes at different developmental stages is required. The mean cell volumes for GV and MII bovine oocytes in isotonic medium were 8.17 × 10^5^ and 7.40 × 10^5^ μm^3^, respectively; accordingly, the surface area was 4.22 × 10^4^ and 3.95 × 10^4^ μm^2^.

### Statistical analysis

Statistical tests were performed using the statistical package R, Version R 3.4.4. The normality of data distribution was checked using the Shapiro–Wilk test and homogeneity of variances through the Levene test. When required, data were linearly transformed into √ x, arcsin √ x or log(x) prior to running statistical tests. An one-way analysis of variance (ANOVA) followed by a pairwise comparison test (Tukey–Kramer adjustment) was used to assess differences molarities within the same CPA and nuclear stage and differences in water or solute permeability between GV and MII stage within the same CPA. Significance was set at p ≤ 0.05.
